# 850. Before-and-After Adoption of a New Fluid Resuscitation Protocol - Parkland Formula versus ABA Consensus Formula

**DOI:** 10.1093/jbcr/irag033.320

**Published:** 2026-04-06

**Authors:** Larissa Wietlisbach, Gabrielle Keller Goff, Annemarie O'Connor, Lawrence Gottlieb, Sebastian Q Vrouwe

**Affiliations:** University of Chicago Burn Center, Chicago, IL; University of Chicago Pritzker School of Medicine, Chicago, IL; University of Chicago Burn Center, Chicago, IL; University of Chicago, Chicago, IL; University of Chicago Burn Center

## Abstract

**Introduction:**

Through much of modern burn care, the “Parkland Formula” was the most common fluid resuscitation protocol in use, where 4 mL/kg/%TBSA is given in the initial 24 post-injury. Concerns regarding over-resuscitation and associated complications led to a shift towards initiating fluids at lower rates. The American Burn Association (ABA) “Consensus Formula” recommends 2 and 3 mL/kg/%TBSA for adults and children, respectively. Our center was an early adopter of this change in practice, and the aim of this study is to compare fluids administered and associated outcomes before and after the adoption of the Consensus Formula.

**Methods:**

Patients who presented to an ABA-verified urban center with burn injuries requiring fluid resuscitation (≥20% and ≥ 15% TBSA for adults and children, respectively) between April 1, 2009 to March 31, 2024 were included. Patients were excluded if they had known kidney disease or were made comfort care in the first week post-injury. A retrospective chart review was performed before and after October 2016, at which time there was a formal transition from the Parkland Formula to the Consensus Formula. Patient and injury characteristics, fluid data, and burn outcomes were compared by protocol group with appropriate statistical tests, and univariate regression analysis was used to identify risk factors for outcomes. Statistical significance was set at *p*<.05.

**Results:**

Seventy-three patients were included in this study, 45 in the Parkland period, and 28 in the Consensus period. There were no significant differences in age, % TBSA, and rate of inhalation injury. At 24 hours, the Parkland group received a mean 4.6 mL/kg/%TBSA and the Consensus group received a mean 3.8 mL/kg/%TBSA (*p*=.791). In terms of complications, there were no differences in the rates of extremity (n = 5, 11% vs n = 4, 14%), abdominal (n = 1, 2% vs n = 2, 7%) or orbital compartment syndromes (n = 0 vs n = 0) between groups. There were no differences in the rates of early acute kidney injury (n = 8, 22% vs n = 4, 15%) or the need for renal replacement therapy (n = 0, 0% vs n = 1, 4%) between groups. Average ventilator days were higher in the Parkland group (42.5 vs 19.9 days, *p*<.05). In-hospital mortality was lower in the Parkland group (n = 2, 4% vs n = 6, 21%; *p*=.024), but there was no significant difference in mortality at one week (n = 0, 0% vs n = 1, 4%).

**Conclusions:**

In this study, the transition from the Parkland to the ABA Consensus formula did not significantly reduce 24-hour fluid volumes or complications of over-resuscitation, nor did it increase the incidence of acute kidney injury. Both groups on average received more than their respective protocols predicted. These findings highlight the persistent challenges of optimizing resuscitation strategies and the need for further prospective studies to refine fluid management in major burn patients.

**Applicability of Research to Practice:**

Regardless of fluid protocol used, burn care providers should remain vigilant of over-resuscitation in burn patients.

**Funding for the study:**

N/A.

